# Breast Cancer Screening and Prophylactic Mastectomy for High-Risk Women in Romania

**DOI:** 10.3390/medicina60040570

**Published:** 2024-03-30

**Authors:** Claudiu Ioan Filip, Andreea Cătană, Eniko Kutasi, Sara Alexia Roman, Mariela Sanda Militaru, Giulia Andreea Risteiu, George Călin Dindelengan

**Affiliations:** 1 Department of Plastic Surgery and Burn Unit, Emergency District Hospital, 400535 Cluj-Napoca, Romania; filip_claudiu2000@yahoo.com (C.I.F.); george.dindelegan@umfcluj.ro (G.C.D.); 2 First Surgical Clinic, Faculty of Medicine, University of Medicine and Pharmacy “Iuliu Hatieganu”, 400012 Cluj-Napoca, Romania; 3 Department of Molecular Sciences, Faculty of Medicine, University of Medicine and Pharmacy “Iuliu Hatieganu”, 400012 Cluj-Napoca, Romania; catanaandreea@gmail.com (A.C.); enikokutasi@gmail.com (E.K.); roman.sara.alexia@elearn.umfcluj.ro (S.A.R.); risteiu.giulia.andreea@elearn.umfcluj.ro (G.A.R.); 4 Department of Oncogeneticcs, Institute of Oncology, “Prof. Dr. I. Chiricuță”, 400015 Cluj-Napoca, Romania; 5 Regional Laboratory Cluj-Napoca, Department of Medical Genetics, Regina Maria Health Network, 400363 Cluj-Napoca, Romania

**Keywords:** breast cancer, prophylactic mastectomy, genetic factors, BRCA, high risk

## Abstract

Breast cancer remains a significant contributor to morbidity and mortality within oncology. Risk factors, encompassing genetic and environmental influences, significantly contribute to its prevalence. While germline mutations, notably within the BRCA genes, are commonly associated with heightened breast cancer risk, a spectrum of other variants exists among affected individuals. Diagnosis relies on imaging techniques, biopsies, biomarkers, and genetic testing, facilitating personalised risk assessment through specific scoring systems. Breast cancer screening programs employing mammography and other imaging modalities play a crucial role in early detection and management, leading to improved outcomes for affected individuals. Regular screening enables the identification of suspicious lesions or abnormalities at earlier stages, facilitating timely intervention and potentially reducing mortality rates associated with breast cancer. Genetic mutations guide screening protocols, prophylactic interventions, treatment modalities, and patient prognosis. Prophylactic measures encompass a range of interventions, including chemoprevention, hormonal inhibition, oophorectomy, and mastectomy. Despite their efficacy in mitigating breast cancer incidence, these interventions carry potential side effects and psychological implications, necessitating comprehensive counselling tailored to individual cases.

## 1. Introduction

Breast cancer (BC) remains the most common type of cancer diagnosed in women. Although in recent decades, there have been fewer cases diagnosed in advanced, metastatic stages, breast cancer remains a major public health issue worldwide [1].

One important characteristic of breast cancer lies in the multiple subtypes that have been described, each presenting its challenges in terms of diagnosis and treatment. These subtypes, from hormone receptor-positive to HER2-positive and triple-negative, guide patient management and prognosis. A complete and correct diagnosis involves understanding the molecular mechanisms behind these subtypes, and it is essential for developing targeted therapies and improving patient outcomes [2].

There are multiple risk factors known for breast cancer besides gender and age, including genetic predisposition, hormonal influences, environmental exposures, and lifestyle variables. Defining these predisposing factors is vital in understanding the aetiology and devising personalised prevention strategies. Treatment options vary from conventional approaches, such as surgery, chemotherapy, and radiotherapy, to immunomodulation and targeted therapy.

Prophylactic measures are essential factors in limiting the incidence of BC, especially for individuals at high risk due to genetic predispositions. Prophylactic mastectomy stands as one of the most important measures, but due to its significant psychological implications, the decision to undergo this type of intervention is often complex. This procedure has become more important in patient management over the past few years. A mastectomy is often followed by reconstructive procedures, which can significantly reduce the psychological impact on a patient [3].

Although there is continuous progress regarding the treatment options in oncology, the prevention and early detection of cancers still represent the key elements for ensuring better outcomes for patients, together with the necessary access to rehabilitation methods and psychological supports in a multidisciplinary approach.

## 2. Prevalence

Epidemiological data provided by GLOBOCAN (2018 and 2020) [4] suggest an increasing incidence of BC. If in 2018 approximately 2.1 million cases were diagnosed, in 2020, the number increased to 2.3 million. The latest report describes breast cancer as the primary type of cancer diagnosed (11.7%), surpassing lung cancer (11.4%), considering both sexes. Mortality rates have been higher in less-developed countries [4,5,6].

Breast cancer exhibits distinct epidemiological characteristics in East European countries, presenting both challenges and opportunities for public health interventions. It represents a significant health concern in Romania, with notable prevalence and associated mortality rates. The incidence of breast cancer in Romania has been on the rise, reflecting a global trend; in 2020, it was 12.2% for both sexes and 26.9% for women. The mortality rates for BC were reported to be 7.2%. The region faces a unique combination of risk factors, including genetic predispositions, lifestyle choices, and socio-economic factors that contribute to the prevalence of breast cancer. Late-stage diagnoses are unfortunately common, often due to limited access to screening programs and healthcare resources. Additionally, certain East European countries may face healthcare infrastructure challenges, impacting early and effective treatment. The burden of breast cancer in these nations is also influenced by cultural perceptions, which may affect prevention efforts, awareness, and the seeking of medical care [7,8,9,10].

Despite the well-known benefits and the current practice in most countries in the EU, genetic diagnosis is not covered by the national health system in Romania, thus making genetic testing and proper diagnosis significantly more difficult for patients with low incomes. Furthermore, Romania lacks a national register in which diagnosed genetic mutations can be reported, and so proper statistical analysis can be performed on the genotypic characteristics of the Romanian population [9].

Our country also needs medical professionals specializing in oncological genetics, and genetic counselling is only available for needy patients. Multidisciplinary boards to discuss oncological cases, especially with a significant genetic background, are available in very few institutions, affecting proper patient care [9].

The public health system is often overwhelmed, especially in the bigger hospitals, since the smaller ones do not have the proper equipment to offer cancer treatment. Although progress in the field is sometimes achieved more quickly, access to the private health system is often limited, implying more significant costs for the patients [9].

## 3. Risk Factors

### 3.1. Genetic Risk Factors in Breast Cancer

Hereditary breast cancers represent approximately 5–10% of all diagnosed breast cancers [11]. In approximately half of the hereditary cases, the defect is inherited from one of the parents according to the dominant model; in the other half, the defect is secondary to a de novo mutation [12], and no significant family histories are reported in these cases. On the other hand, a positive family history is not necessarily synonymous with the carrier status of germline mutations. Polygenic determinism, characterised by multiple molecular defects with additive, convergent effects accompanied by other risk factors, also plays a role in familial aggregation and cancer development [12,13,14].

#### 3.1.1. BRCA Mutations

BRCA1 and BRCA2 play crucial roles as tumour suppressors in repairing double-strand DNA breaks (induced by natural and medical-use radiation or other environmental hazards) through the process of homologous recombination (HR) [15]. Aberrant BRCA1 and BRCA2 activities lead to the accumulation of mutations and abnormal cell divisions. Although most of these cells are genetically unstable and do not survive, some acquire malignant potential, thus leading to tumour formation. BRCA1 is also involved in cell-cycle regulation, transcription, and chromatin remodelling [16].

The role of the BRCA2 gene is to regulate the activity of *RAD51*, another gene involved in the DNA repair process [16,17]. Both BRCA genes are associated with a high risk of breast and ovarian cancer, with most of the BC cases being non-special-type ductal carcinomas [18]. The most common histopathological subtype associated with BRCA1-positive breast cancers is typically the “basal-like” or “triple-negative” subtype. Triple-negative breast cancer (TNBC) is characterised by the absence of estrogen receptor (ER), progesterone receptor (PR), and human epidermal growth factor receptor 2 (HER2) expression. The basal-like subtype shares similarities with triple-negative breast cancer and is characterised by the expression of basal cytokeratins (such as CK5/6 and CK14). These tumours often have a high histological grade, pushing margins, and a higher likelihood of presenting lymphocytic infiltrates [17,18,19].

DNA repair mechanism deficiencies make cells more susceptible to specific therapeutic agents. Poly ADP-ribose polymerase (PARP) inhibitors have emerged as a promising class of drugs for *BRCA*-associated cancers. These inhibitors exploit the impaired DNA repair in *BRCA*-mutated cells, leading to enhanced cancer cell death. BRCA-associated breast and ovarian cancers have been shown to be more sensitive to specific chemotherapeutic agents, such as platinum-based drugs. This knowledge can guide treatment decisions and optimise therapeutic outcomes [20,21,22].

As patients with *BRCA* mutations present significantly higher risk for breast cancer development, enhanced surveillance methods and proper genetic counselling are necessary. Prophylactic measures may include double mastectomy and oophorectomy [23,24].

#### 3.1.2. Non-BRCA Mutations

Other high-penetrance genes associated with breast cancer have been described, such as *TP53* (Li-Fraumeni syndrome), *PTEN* (Cowden syndrome), *PALB2*, and *STK11* (Peutz-Jeghers syndrome) [24]. Pathogenic germline mutations in these genes contribute to an increased risk of various cancers, including breast cancer.

*PALB2* (a partner and localiser of *BRCA2*) plays a crucial role in DNA repair. *PALB2* mutations impair the function of the BRCA protein complex, leading to compromised DNA repair mechanisms and increased genomic instability. Biallelic germline loss-of-function mutations in the *PALB2* gene can lead to Fanconi’s anemia [25], while monoallelic impairments increase the risk of breast and pancreatic cancer [26,27]. *PALB2* mutations are often associated with the development of the triple-negative breast cancer (TNBC) subtype, similar to *BRCA1* mutations. Similar to *BRCA* mutations, *PALB2*-associated breast cancers may exhibit sensitivity to PARP inhibitors. Additionally, understanding the molecular subtypes guides therapeutic decisions, as TNBC often requires tailored treatment approaches, such as chemotherapy [26,27].

*TP53*, known as the “guardian of the genome”, regulates cell cycle progression and prevents the growth of cells with damaged DNA. Thus, mutations result in the loss of this tumour-suppressive function, contributing to uncontrolled cell proliferation. Somatic acquisitions in the *TP53* gene are the most observed alterations in cancer patients, occurring in approximately 30% of all breast cancer cases. Carriers of germline mutations in the *TP53* gene have a risk of breast cancer of up to 85% by the age of 60 years. Most of these breast cancers are early onset, with the median age at diagnosis being 34 years [28].

*TP53* mutations are observed across various breast cancer subtypes, including luminal B and HER2-positive, but they are more commonly associated with the basal-like subtype. Breast cancers with *TP53* mutations may resist multiple available therapies, and treatment decisions need to consider specific molecular characteristics [28,29]. The overall prognosis for these patients is poor compared to those with wild-type *TP53* [29].

Germline mutations in the *PTEN* gene are associated with Cowden syndrome, a hereditary hamartomatous syndrome with a heterogeneous phenotype. *PTEN* acts as a tumour suppressor by regulating division and cell growth. *PTEN* mutations result in uncontrolled cell growth and contribute to cancer development. Thus, they may be involved in the development of diverse breast cancer subtypes, including hormone receptor-positive and HER2-positive subtypes. The lifetime risk with a *PTEN* mutation is approximately 40–60% compared to 12.5% for the general population. *PTEN*-mutated breast cancers may exhibit altered signalling pathways, impacting responsiveness to targeted therapies, but other therapies targeting molecular pathways, such as PI3K inhibitors, represent an area of active research [30,31].

E-cadherin (*CDH1* gene) germline mutations are more often linked to diffuse gastric cancer, but they also come with an inherited predisposition to develop lobular breast carcinoma (LBC). *CDH1* encodes E-cadherin, a cell adhesion molecule. *CDH1* mutations disrupt cell adhesion, promoting invasiveness and metastasis. Prophylactic measures, such as preventive mastectomy, may be considered for individuals with *CDH1* mutations [32,33].

The ataxia-teleangiectasia mutated (*ATM*) gene has a prevalence of 40% in BC patients. Pathogenic germline variants of *ATM* are associated with an increased risk of BC [34] and a worse prognosis since the tumour itself is more aggressive. There is a higher rate of lymph node involvement. *ATM* missense variants have a similar effect as *BRCA1* mutations on cancer cells, sensitizing the cancer cells to platinum-derived drugs. There seems to be a higher risk of chemo- and radio-therapy resistance and of developing secondary tumours in both breasts [35].

In addition to high-penetrance genes, researchers have identified several moderate and low-penetrance genes that influence breast cancer risk. Germline defects in the RAD51, CHEK2, and BARD1 genes may have a more subtle impact individually but can collectively contribute to an increased susceptibility [11,12,13,14].

The genetic characteristics of hereditary BC in Romania do not differ much from those reported after observing the Slavic and East Caucasian populations. *BRCA* mutations and a positive family history of cancer are linked to earlier diagnoses of BC compared to other mutations. Non-*BRCA* mutations have been described most frequently in the *CHEK2*, *ATM*, and *PALB2* genes [36].

### 3.2. Non-Genetic Risk Factors

Breast cancer is associated with various risk factors, such as female sex and advanced age. Among the modifiable risk factors, the most important are obesity, chronic consumption of oral contraceptives and hormone replacement therapies, radiation exposure, alcohol consumption, smoking, a diet rich in saturated fats and synthetic sugars, and lack of physical exercise [37].

The early onset of menstruation and late menopause, advanced reproductive age, and lack of breastfeeding can also cause prolonged exposure to higher estrogen levels and, thus, a higher risk of developing BC. Variations in breast tissue density and architecture contribute to the complexity of breast cancer risk stratification. Dense breast tissue, characterised by a higher proportion of glandular and fibrous tissue relative to fatty tissue, is recognised as a risk factor for breast cancer. Dense breasts not only make mammographic detection more challenging but are also associated with an increased likelihood of developing cancer. This heightened risk is attributed to the increased cellular activity and potential for developing abnormal cells within denser tissue. Conversely, breasts with a higher proportion of fatty tissue tend to have a lower cancer risk [38,39,40,41].

### 3.3. Risk Stratification

Several breast cancer risk assessment models are used in clinical practice and research, and we will focus on some of the more commonly used ones.

The Gail Model is a widely employed tool for estimating the risk of developing invasive breast cancer in women. It calculates risk based on several factors, including age, race, age at menarche, age at first live birth, number of first-degree relatives with breast cancer, and the presence of atypical hyperplasia on breast biopsy. The Gail Model provides a 5-year and lifetime risk estimate, aiding in clinical decision-making regarding screening and preventive interventions. The simplicity and accessibility of the Gail Model contribute to its extensive use in both research and clinical settings [42].

An updated version of the Gail Model, the Breast Cancer Risk Assessment Tool (BCRAT), incorporates additional risk factors to enhance precision. It includes race/ethnicity, personal history of breast cancer or ductal carcinoma in situ (DCIS), and certain benign breast diseases as part of its risk calculation. As with its predecessor, the BCRAT provides 5-year and lifetime risk estimates. Its online availability through the National Cancer Institute’s website facilitates widespread use. The BCRAT is particularly valuable in assessing risk in diverse populations and aids in identifying individuals who may benefit from intensified screening or preventive measures [43].

The Tyrer–Cuzick model, also known as the International Breast Cancer Intervention Study (IBIS) model, is a comprehensive risk assessment tool that incorporates many risk factors. It calculates breast cancer risk by considering factors such as age, family history of breast cancer, hormonal factors (e.g., age at menarche and age at first birth), breast density, and the presence of specific genetic mutations (e.g., BRCA1 and BRCA2). This model is precious for assessing risk in women with a family history of breast cancer and provides a more refined risk estimate. The IBIS model aids in personalised risk management and decision-making related to surveillance and preventive measures [44].

## 4. Investigations

Systematic exploration consists of documenting the clinical presentation, imagistic characteristics, histological variations, molecular genetics, and specific biomarkers, each contributing to the comprehension of the underlying pathophysiology [45].

### 4.1. Germline Genetic Testing

As breast cancer often exhibits familial patterns, germline testing is a key component of genetic counselling and precision medicine. The focus primarily lies in identifying pathogenic variants in specific genes, such as *BRCA1*, *BRCA2*, *TP53*, *PTEN*, and others, which have been associated with hereditary breast cancer predisposition. These genetic mutations, when present in the germline, significantly elevate the lifetime risk of developing breast cancer [15,16,24,28].

Identifying the carriers of pathogenic variants is valuable for affected individuals and their family members who may also be at risk. The early detection of these mutations enables risk reduction and tailored screening strategies. Germline testing can also influence treatment decisions. High-risk individuals may opt for preventive measures, such as enhanced screening, prophylactic surgeries, and lifestyle modifications, to mitigate their risk of breast cancer [17,25,29].

The revelation of hereditary risk may have profound emotional and social implications. As such, comprehensive genetic counselling is an integral component of germline testing, providing individuals and their families with the necessary information and support to make informed decisions [24].

### 4.2. Imaging

Imaging methods are essential both as screening options and as part of the diagnostic procedures for breast cancer. The screening methods rely on mammary ultrasounds at a younger age and mammography later in life, when the breast tissue is better seen with this method.

Ultrasounds are used to distinguish between solid and cystic masses and guide the biopsies once the tumours are found. Mammography is highly effective in finding early signs of cancer, such as microcalcifications, especially in asymptomatic cases. Three-dimensional mammography enhances diagnostic accuracy. Molecular breast imaging (MBI) can be more effective for scanning dense breast tissue [46].

MRIs provide detailed images of any potential lesions and can also evaluate the extent of the disease or monitor the response to neoadjuvant therapy. CT and PET-CT are especially useful in assessing the extent of the disease’s spread.

While still an evolving technology, thermography has been explored for its potential as a screening tool. It measures temperature variations in breast tissue, and although it is not a standalone diagnostic tool, it may provide additional information for further evaluation [47,48].

In Romania, there have been some attempts to increase health literacy and the general population’s access to mammography as a screening procedure covered by national health programs and through projects implemented by NGOs, but the impact remains very low. Recent studies show that 79% of the women at risk from Romania have never undergone mammographies for breast cancer screening [9].

### 4.3. Biological Markers

Biomarker assessment in breast cancer plays a crucial role in proper diagnosis, predicting prognosis, and guiding treatment decisions. The presence or absence of an estrogen receptor (ER) and a progesterone receptor (PR) indicates a tumour’s hormone receptor status, predicting the response to hormonal therapies. Hormone receptor-positive tumours may respond to endocrine therapies such as tamoxifen or aromatase inhibitors [49].

Human Epidermal Growth Factor Receptor 2 (HER2) is a protein that promotes cancer cell growth. Thus, HER2-positive breast cancers are associated with aggressive behaviour, but they can be targeted with therapies such as trastuzumab (Herceptin). Tumours lacking both hormone receptors and HER2 are called triple-negative [49].

Ki-67 is a marker of cell proliferation and tumour aggressiveness and can predict the response to chemotherapy [49,50].

CDK4/6 amplification can also be evaluated in hormone receptor-positive breast cancers. Cyclin-dependent kinase 4/6 (CDK4/6) inhibitors, such as palbociclib and ribociclib, target cell-cycle regulation [50].

Serum markers, such as CA 15-3 and CA 27.29, while not diagnostic on their own, aid in monitoring disease progression and treatment response in advanced stages.

These biomarkers altogether enable personalised treatment approaches and provide critical insights into the biological characteristics of breast cancer [49,50,51].

## 5. Prophylactic Measures

Together with controlling the modifiable risk factors, self-assessment as part of a rigorous screening protocol should be practised by all women, documenting any masses that occur and are not related to regular modifications during the menstrual cycle. A specialist should assess any palpable mass to rule out the presence of a malignant tumour [47].

A study conducted in Romania assessed the rates of self-examination for breast cancer screening to measure medical literacy on this topic. Meagre rates of monthly self-examination were reported in the age groups of 15–24 and above 50, marking these groups as vulnerable. The most cases of correct self-examination were described in the age group of 25–49 [52].

### 5.1. Chemoprevention and Hormonal Inhibition

Chemoprophylaxis involves the use of certain drugs or natural compounds to reduce the risk of cancer occurrence or its progression [53]. At a cellular and molecular level, it aims to control protein activities during cancer development initiation, promotion, or progression stages. Ball et al. collected data on the following main chemoprevention methods: selective estrogen receptor modulators (SERMs), aromatase inhibitors, and aspirin [53,54]. 

SERMs are the most used chemoprevention method among high-risk women. They work by modulating the estrogen response. The prominent representatives of this class are Tamoxifen, Raloxifen, and Lasofoxifene [54,55,56,57,58,59]. The National Surgical Adjuvant Breast and Bowel Project P-1 (NSABP-P1) study proved that Tamoxifen use (20 mg/day for five years) led to a 49% risk reduction in invasive breast cancer compared with a placebo, with even higher risk reductions observed in elders [60].

Comparatively, Raloxifene was proven to be equally effective as Tamoxifene in decreasing the risk of invasive breast cancer [55,56,57]. Lasofoxifene is a third-generation SERM with greater potency. Studies have shown that Lasofoxifene can reduce the risk of total BC incidence by 79% and ER+ invasive breast cancer incidence by 83% compared to a placebo administration [59,60].

Raloxifene has been associated with a minor risk of developing thromboembolic events, whereas the incidences of ischemic heart disease, stroke, and osteoporotic fractures were similar for both Tamoxifen and Raloxifene. Endometrial cancer was reported less frequently in association with Raloxifene use compared to Tamoxifen use [58,59,60,61].

Aromatase inhibitors, such as Exemestane and Anastrozole, are primarily used by postmenopausal women. They inhibit the production of estrogen, which can promote certain types of breast cancers, by inhibiting the aromatase enzyme. As potential side effects, aromatase inhibitors may lead to bone density loss and musculoskeletal issues [62,63].

Aspirin may also have chemopreventive effects against breast cancer. While relatively safe, it can also have side effects such as gastrointestinal bleeding [64].

Multiple natural phenolic compounds have been proven to play a potential role in breast cancer chemoprevention by modifying several epigenetic factors involved in carcinogenesis [65].

Curcumin and its analogues can represent potential chemoprevention agents by exerting their antiproliferative and anti-inflammatory properties [66]. Diindolylmethane, a bioactive compound found in cruciferous vegetables, promotes changes in estrogen metabolism and may also aid in chemoprophylaxis [67].

While chemoprevention can bring benefits in preventing breast cancer development, it is essential to weigh the potential benefits against the risks and side effects of these agents.

### 5.2. Oophorectomy

Oophorectomy, also known as ovarian removal or ovariectomy, is a surgical procedure which implies the removal of one or both ovaries. This procedure can have significant implications for breast cancer prevention, particularly in women who are considered at high risk of developing breast cancer due to genetic or other risk factors [68,69].

The primary goal of oophorectomy in this context is to reduce estrogen and progesterone levels, which can stimulate the growth of hormone receptor-positive breast cancer cells. Risk-reducing bilateral salpingo-oophorectomy (RRBSO) is recommended by international guidelines for healthy women carrying germline *BRCA1* and *BRCA2* mutations, starting at the age of 35–40 years for *BRCA1* and 40–45 years for *BRCA2* mutation carriers. The intervention is recommended after proper family planning because of its consequent premature menopause. RRBSO potentially causes other side effects related to estrogen deprivation [70].

While it reduces the risk of developing breast cancer without eliminating it, the decision to undergo an oophorectomy is complex, and it involves significant psychological challenges, especially for young women [71].

It is essential for individuals considering oophorectomy to discuss their fertility preservation options and concerns with their healthcare providers. In the case of a unilateral oophorectomy (the removal of one ovary), the remaining ovary may continue to produce eggs, preserving fertility to some extent. However, a bilateral oophorectomy (the removal of both ovaries) results in the loss of egg production, rendering a woman unable to conceive naturally. The hormonal changes which follow this procedure may also impact fertility. Before undergoing an oophorectomy, a woman who wishes to preserve her fertility may explore options such as oocyte (egg) or embryo cryopreservation, which involves harvesting and freezing eggs or embryos for future use in assisted reproductive technologies such as in vitro fertilization (IVF) [72].

### 5.3. Mastectomy

The optimal surgical management of BRCA-mutation carriers remains a debatable subject. Although surgical prophylaxis is the preferred option in a majority of the BRCA-positive cases, other studies suggest that BRCA mutation carriers treated with BCT present similar oncological outcomes compared to mastectomy, and young BRCA patients with incipient BCs may not need up-front mastectomy, though prophylactic surgery might be performed when ovarian cancer risk epidemiologically rises and potential reproductive desire is fulfilled [73].

Prophylactic mastectomies, preemptive surgical measures for those at heightened risk of breast cancer, involve diverse techniques tailored for risk reduction and aesthetic outcomes [72].

A bilateral mastectomy, the removal of both breast tissues, offers options such as simple mastectomy, skin-sparing mastectomy (preserving skin for future reconstruction), and nipple-sparing mastectomy (preserving the nipple-areola complex). A contralateral prophylactic mastectomy (CPM) addresses the unaffected breast in the case of unilateral breast cancer, employing techniques similar to those used in a bilateral mastectomy [74,75].

Immediate breast reconstruction frequently accompanies a prophylactic mastectomy, presenting choices such as autologous tissue (e.g., DIEP flap) or implants. This reconstruction may co-occur with the mastectomy or during a later intervention. Individuals carrying *BRCA1* or *BRCA2* mutations, associated with elevated breast cancer risk, often opt for risk-reducing mastectomies, significantly diminishing the likelihood of future breast cancer occurrences [76,77,78].

Emerging techniques include robotic-assisted surgery for precision, especially in nipple-sparing mastectomies, and oncoplastic approaches that blend aesthetics with risk reduction [79].

Prophylactic mastectomy, while a potent risk reduction strategy, profoundly affects individuals psychologically. The decision involves complex considerations, impacting body image, self-esteem, and intimate relationships. Emotional responses vary, encompassing anxiety, grief, and relief. Decision-making stress is common, and post-surgical adjustment takes time [80,81].

Genetic counselling is integral to the decision-making process. It provides individuals with a comprehensive understanding of their genetic risk, the implications of testing positive for specific mutations, and potential alternatives to prophylactic mastectomies. Counselling offers a supportive space for discussing the psychological aspects, allowing individuals to make informed decisions aligned with their values and preferences [82,83].

In Romania, there are only a few professionals who specialise in both oncological and reconstructive surgery. Thus, access to both proper treatment and aesthetic results is often difficult.

#### Psychological Impact of a Mastectomy

Women with mastectomies have shown high satisfaction rates, reaching 70% after 14.5 years from a bilateral mastectomy and ranging between 83% and 90% after 10.3–20 years from contralateral mastectomies [84]. However, positive body image was significantly affected, especially with bilateral mastectomies, due to many factors, such as self-consciousness, feeling less sexually attractive, and dissatisfaction with the scars. Decreased sexual satisfaction was linked to both body image issues and a loss of sensation in the breast [85,86].

Women who underwent unilateral mastectomies were less satisfied with their appearance than those who underwent bilateral mastectomies. Some data suggest that reconstruction is associated with lower long-term satisfaction, explained by more frequent surgical complications and concerns about implants [87]. Ha et al. studied insurance coverage across the United States and discovered the following: Preauthorised coverage for prophylactic mastectomies was assured by 39% of insurance policies (n = 39). There was a consensus amongst these policies to cover prophylactic mastectomies for *BRCA1* and *BRCA2* mutations (n = 39, 100%), but the coverage was variable for other genetic mutations (15–90%). In the United Kingdom, according to the NHS, prophylactic mastectomies are eligible without additional funding for all high-risk women [88,89].

Studies conducted in Romania, which have included women diagnosed with breast cancer as a target group, have shown that support groups and psychotherapy offered both before and after undergoing treatment were very helpful in improving a patient’s mental health status and overall quality of life. Furthermore, a better quality of life was described even following online group sessions, which implied open discussions about their fears and the diagnosis’s impact on their personal and social lives. Thus, the psychological impact of this diagnosis appears to be reduced by proper psychological support, to which breast cancer patients in Romania have access in some instances [90].

## 6. Conclusions

Breast cancer remains one of the leading causes of morbidity and mortality among oncological pathologies. Various risk factors have been linked to a higher prevalence of breast cancer, among which genetic and environmental factors play essential roles.

Germline mutations associated with higher risk for BC are most commonly found in the *BRCA* genes, but various other variants have been described in affected individuals. The diagnoses rely on imaging methods, biopsies, biological markers, and genetic testing. The cancer risk for each individual can be assessed using specific scores and evaluations.

Potential genetic mutations guide the screening protocols and prophylactic measures, as well as the treatment options and prognoses, of breast cancer patients. Prophylactic interventions involve chemoprevention, hormonal inhibition, oophorectomy, and mastectomy. While these measures can help prevent breast cancer development, they also come with potentially significant side effects or psychological implications, and proper counselling is required in each case.

## Data Availability

The data presented in this study are available on request from the corresponding author.
